# Green Synthesis and Characterization of Iron Nanoparticles Synthesized from Aqueous Leaf Extract of *Vitex leucoxylon* and Its Biomedical Applications

**DOI:** 10.3390/nano12142404

**Published:** 2022-07-14

**Authors:** Mohammed H. Nahari, Amer Al Ali, Abdulaziz Asiri, Mater H. Mahnashi, Ibrahim Ahmed Shaikh, Arun K. Shettar, Joy Hoskeri

**Affiliations:** 1 Department of Clinical Laboratory Sciences, Najran University, Najran 66216, Saudi Arabia; mhnahari@nu.edu.sa; 2 Department of Clinical Laboratory Sciences, Faculty of Applied Medical Sciences, University of Bisha, 255, Al Nakhil, Bisha 67714, Saudi Arabia; ameralali@ub.edu.sa; 3 Department of Basic Medical Sciences, Faculty of Applied Medical Sciences, University of Bisha, 255, Al Nakhil, Bisha 67714, Saudi Arabia; amfasiri@ub.edu.sa; 4 Department of Pharmaceutical Chemistry, College of Pharmacy, Najran University, Najran 66216, Saudi Arabia; 5 Department of Pharmacology, College of Pharmacy, Najran University, Najran 66216, Saudi Arabia; i.ibrahimshaikh09@gmail.com; 6 Division of Preclinical Research and Drug Development, Cytxon Biosolutions Pvt Ltd., Hubli 580031, Karnataka, India; aruncytxon9@gmail.com; 7 Department of Bioinformatics and Biotechnology, Karnataka State Akkamahadevi Women’s University, Vijayapura 586108, Karnataka, India; joybioinfo@gmail.com

**Keywords:** *Vitex leucoxylon*, iron nanoparticles, SEM, wound healing, cytotoxic

## Abstract

The cold extraction method was used to obtain the aqueous extract of *Vitex leucoxylon* leaves in a ratio of 1:10. Iron nanoparticles (FeNPs) were synthesized using aqueous leaf extract of *V. leucoxylon* as a reducing agent. The phytoreducing approach was used to make FeNPs by mixing 1 mL of plant extract with 1 mM of ferric sulfate. Scanning electron microscopy (SEM), Fourier-transform infrared spectroscopy (FTIR), Ultraviolet–visible spectroscopy (UV-Vis), and energy-dispersive X-ray spectroscopy were used to examine the synthesized FeNPs. The reducing reaction was shown by a change in the color of the solution, and the formation of black color confirms that FeNPs have been formed. The greatest absorption peak (max) was found at 395 nm in UV-Vis spectral analysis. The FTIR spectra of *V. leucoxylon* aqueous leaf extract showed shifts in some peaks, namely 923.96 cm^−1^ and 1709.89 cm^−1^, with functional groups carboxylic acids, unsaturated aldehydes, and ketones, which were lacking in the FTIR spectra of FeNPs and are responsible for FeNPs formation. FeNPs with diameters between 45 and 100 nm were observed in SEM images. The creation of FeNPs was confirmed by EDX, which shows a strong signal in the metallic iron region at 6–8 Kev. XRD revealed a crystalline nature and an average diameter of 136.43 nm. Antioxidant, anti-inflammatory, cytotoxic, and wound healing in vitro tests reported significant activity of the FeNPs. The cumulative findings of the present study indicate that the green synthesis of FeNPs boosts its biological activity and may serve as a possible dermal wound-healing agent and cytotoxic agent against cancer. Future study is needed on the identification of mechanisms involved in the synthesis of FeNPs by *V. leucoxylon* and its biomedical applications.

## 1. Introduction

The application of nanotechnology in science and technology to manufacture new materials at the nanoscale level is a rapidly growing field [1]. Nanotechnology deals with manufacturing materials at the atomic level to gain distinctive properties, which can be manipulated for preferred applications. This field is rapidly growing with its applications in science and technology to manufacture new materials at the nanoscale level [2]. Various industrial sectors have embraced nanotechnology in recent years due to its applications in the fields of electronic storage systems [3], biotechnology [4], magnetic separation and preconcentration of target analytes, targeted drug delivery [5], and vehicles for gene and drug delivery [3,5,6]. Consequently, these particles have the potential to make a substantial impact on society as a result of the wide range of applications for which they can be used. Nanoparticles (NPs) are masses of particles that have a size less than one hundred nanometers and are regarded to be key structural masses in the field of nanotechnology. The NPs’ higher activity is both their defining characteristic and the quality for which they are most notable [7]. Organic and inorganic NPs are the two primary classifications that can be applied to nanoparticles. Inorganic nanoparticles may include magnetic NPs, noble metal NPs (such as gold and silver), and semiconductor NPs (such as titanium dioxide and zinc oxide). Organic nanoparticles may include carbon NPs. Inorganic nanoparticles are attracting huge attention because they offer superior material properties along with functional versatility. They have been investigated as possible tools for medical imaging as well as for the treatment of diseases due to the size characteristics they possess [8]. The diverse chemical, physical, and biological properties of NPs are heavily influenced by a variety of parameters, including nanoparticle size and morphology, as well as surface coating, which are normally determined during synthesis of nanoparticles. The loss of intended biological activity is caused by a decrease in colloidal stability. pH, ionic strength, and a slew of proteins that interact with AgNPs under biorelevant circumstances all have an impact on colloidal stability. As a result, proper synthesis technique selection is critical for obtaining the desired particle characteristics for specific applications [9].

Currently, there is a wide variety of NPs that can be synthesized using a variety of physical, chemical, biological, and hybrid processes. According to the findings of a large number of research studies, the physical and chemical processes for producing nanoparticles include the use of organic solvents, hazardous compounds, significant amounts of energy, and stabilizing agents that are not biodegradable [10]. Therefore, in the field of green nanotechnology, the synthesis of NPs using naturally available materials such as plant extracts, various microorganisms, their metabolites, and a few natural humic substances [11,12] as reducing and capping agents is becoming increasingly popular. Synthesis methods that are friendly to the environment have been used to produce a wide variety of metallic nanoparticles, including silver, gold, iron, copper, and zinc. The development of easy and environmentally acceptable methods for the synthesis of NPs is one of the key focuses of nanotechnology. Biomaterials such as microorganisms and plant extracts can be utilized in the process of preparing a wide variety of NPs [13,14]. However, because some organisms are pathogens, it is dangerous to handle them. In order to thrive, microorganisms need to be maintained in culture and subjected to carefully controlled conditions, including temperature, pH, and other parameters. Because it eliminates the laborious process of maintaining the microbial culture, the synthesis of NPs using plant parts can sometimes prove to be more advantageous than other biological processes [15]. As a result, it has garnered a lot of attention due to its inherent characteristics, which include the utilization of natural resources, rapidity, eco-friendliness, and benignancy. These alluring characteristics are absolutely necessary for use in medical applications. The nanoparticles produced by green synthesis have a size that is well-defined and under control, they are free of pollutants, and the method is simple to scale up. These are some of the additional benefits of green synthesis [16]. The biological activity of the synthesized nanoparticles is largely determined and fine-tuned by the green materials utilized for stability and reduction of metal ions. One of the ideal properties of the NPs should be that it must have an outstanding capacity to discriminate between potential targets (pathogens) and mammalian (host) cells [17].

Because of this, the aqueous extract of *V. leucoxylon* leaves was investigated for its potential to facilitate the formation of iron nanoparticles (FeNPs) in the current research. Iron is one of the elements that can be found in the greatest abundance on earth. Recently, it has come to be recognized as a new class of important NPs due to the fact that it possesses a variety of unique properties, including high coerciveness and superparamagnetism. Catalysis, electronic devices, information storage, sensors, drug-delivery technology, biomedicine, magnetic recording devices, and environmental cleanup are just some of the many intriguing applications that have made use of FeNPs [18]. Moreover, according to a number of studies, FeNPs may be created from various plant extracts. These plant extracts include *Eucalyptus globulus* leaf [19], pomegranate leaf [20], and banana peel ash [21]. The plant *V. leucoxylon*, which was used in the current study, is a member of the family Verbenaceae. It is also known as the five-leaved chaste tree (Kannada: Sengeni, Holenekki, Hollalakki) and can be found in the region along river banks in evergreen and semi-evergreen forests and moist deciduous forests along streams. It can reach a height of up to 20 m and is classified as a modest to large deciduous tree. Along the length of India’s Western Ghats forests, it can be found in large numbers. *V. leucoxylon* leaf extract infusion has been reported to possess a wide variety of pharmacological activity such as anti-inflammatory, antioxidant, antipsychotic, antidepressant, antiparkinsonian, and antihyperlipidemic activities [22]. Natural products have, throughout history and particularly in folk medicine, been utilized for the treatment of a wide variety of ailments and illnesses. This practice dates back to ancient times. The methods of natural product chemistry that have been around for a long time have made it possible to find a huge variety of bioactive secondary metabolites that come from terrestrial and marine sources. A significant number of these naturally occurring substances are now being considered for use as potential pharmaceuticals [23].

There are a great number of naturally occurring chemicals and nutrients that have yet to be uncovered that are useful to humanity. As a consequence of this, there is an immediate demand for research and development of innovative therapeutic options that may be utilized successfully in therapeutic interventions while creating a minimum amount of adverse effects.

The currently suggested green synthesis process for FeNPs is distinct and cost-effective. In the current study, an attempt was made to create nanoparticles at room temperature without the use of any chemicals or physical techniques. The *Vitex leucoxylon* plant was chosen in an effort to synthesize iron nanoparticles, and comprehensive systematic in vitro models were carried out to assess the potency of iron nanoparticles. Only a limited number of research studies on the concept of nanoparticles and their biomedical applications have been published on this plant. As a consequence, we decided that it would be beneficial to carry out this research with the following goals in mind: screening for phytochemicals and measuring the amount of secondary metabolites in *V. leucoxylon*; green synthesis and characterization of FeNPs from *V. leucoxylon*; comparative study of antioxidant and anti-inflammatory effects of *V. leucoxylon* and its FeNPs in vitro; in vitro cytotoxic activity of aqueous leaf extract of *V. leucoxylon* and its FeNPs against skin cancer, lung cancer, and oral cancer; in vitro wound-healing activity of aqueous leaf extract of *V. leucoxylon* and its synthetic FeNPs by scratch assay.

## 2. Materials and Methods

### 2.1. Collection of Plant Material

In the month of March 2022, fresh leaves of *V. leucoxylon* were picked from the Anshi forest area of the Western Ghats in the Uttar Kannada District of the state of Karnataka in India. The leaves were identified and authenticated by Dr. Kotresha K., Taxonomist, Department of Botany, Karnataka Science College, Dharwad; Karnataka, by referring to the voucher specimen deposited in the Department of Botany, Karnataka Science College, Dharwad, Karnataka. After being collected, the fresh plant leaf material was washed under running tap water, sun-dried, and then ground into a coarse powder using a mechanical grinder. The powder was kept in containers that were sealed at room temperature so that it could be used later in the process of crude solvent extraction.

### 2.2. Preparation of Plant Extract

Using a Soxhlet device, 25 g of powdered leaves was extracted for 48 h with 250 mL of distilled water. The aqueous extract was concentrated further using a roto-evaporator, and was then dried in desiccators before being kept in an enclosed bottle at 4 °C until use. For the synthesis of FeNPs, the aqueous extract was utilized as a reducing and stabilizing agent.

### 2.3. Solvents and Reagents

All of the employed chemicals and solvents were of analytical quality and were purchased from Hi-media (Hubli, India).

### 2.4. Phytochemical Analysis

Following the procedure described by Deepti et al. (2012), the crude aqueous leaf extract *V. leucoxylon* was qualitatively tested for the presence of various phytochemical constituents such as flavonoids, alkaloids, phenols, glycosides, sterols, lignins, saponins, anthraquinones, tannins, and reducing sugars [24].

### 2.5. Synthesis of Iron Nanoparticles

First, 1 mL of *V. leucoxylon* aqueous leaf extract was added to 10 mL of 0.05 mM FeSO4 aqueous solution, and the mixture was shaken. At room temperature and in a dark environment, the complete reaction mixture process was carried out. The oxidation/reduction reaction was clearly visible after the colorless reaction mixture had been incubated and reacted for the required amount of time [25]. In order to remove any traces of aqueous extract from the freshly synthesized FeNPs, which were allowed to dry in powder after centrifugation at 10,000 rpm for ten 10 min during the desired reaction period, the aqueous mixture containing FeNPs was centrifuged a second time and redispersed in double-distilled water and dried [26]. 

### 2.6. Characterization of FeNPs

Several methods, such as Ultraviolet–visible spectroscopy (UV-Vis), Fourier-transform infrared spectroscopy (FTIR), scanning electron microscopy and energy dispersive X-ray spectroscopy, X-ray Diffraction (XRD), particle size analyzer, and zeta potential, were utilized in order to characterize the FeNPs. 

#### 2.6.1. UV–Visible-Spectroscopy-Based Analysis

First, 1 mL aliquot of colloidal FeNPs solution in quartz cuvettes was evaluated using UV–visible spectroscopy (U-3310, Hitachi, Tokyo, Japan), using distilled water as a reference and 0.05 mM FeSO4 as a blank, to validate the reduction of the ferric ions in the colloidal solution [27].

#### 2.6.2. FTIR-Based Analysis

The function groups (biogroups) that were bound on the iron surface and were involved in the synthesis of FeNPs were identified using FTIR spectroscopy (S700, Nicolet, MA, USA), [28]. After 72 h of incubation, the FeNPs were isolated by repeated centrifugation (3–4 times) of the reaction mixtures at 10,000 rpm for 15 min. The supernatant was replaced by deionized water and the pellet was stored as powder. After being dried, the FeNPs were put through an FTIR analysis using the potassium bromide pelleting process at a ratio of 1:100.

#### 2.6.3. Scanning-Electron-Microscopy-Based Analysis

Scanning electron microscopy (JSM-IT 500, Jeol, Boston, MA, USA), was used to examine the nanoparticles and establish their surface shape. Substrates were prepared on a clean 5 mm × 5 mm Si substrate cleaved from a 100 mm diameter wafer. The substrate was allowed to react for 2 h to 6 h and the sample was prepared by centrifuging a colloidal solution at 10,000 rpm for 5 min. The pellet was dried after being recentrifuged many times, after which it was redispersed in deionized water and the procedure was repeated. Finally, the dry pellet was obtained, which was further subjected to the structural characterization by SEM analysis as per the procedure described by National Institute of Standards and Technology, NIST-2007 [29].

#### 2.6.4. Energy Dispersive X-ray

Following drying on a carbon-coated copper grid, the reduced FeNPs were analyzed using EDX (JSM-IT 500, Jeol, Boston, MA, USA), which also allowed the elemental composition to be determined.

#### 2.6.5. Zeta Potential Observations of NPs

The zeta potential is a useful tool for gaining additional insights on the stability of the colloidal NPs. The amplitude of the zeta potential provides a hint as to the possible stability of the colloid. According to Meléndrez et al. (2010), particles are regarded as being stable if their zeta potential values are either more positive than +30 mV or more negative than 30 mV [30]. This fact should be taken into consideration. The laser zeta meter was utilized in order to acquire readings of the surface zeta potentials (Malvern zeta seizer 2000, Malvern, UK). The liquid samples of the nanoparticles, totaling 5 milliliters, were diluted with 50 milliliters of double-distilled water, and 2 mm per square meter of sodium chloride was used as the suspending electrolyte solution. After that, the pH was modified until it reached the desired level. The samples were agitated for a total of 30 min. After shaking the container, the pH at equilibrium was noted, and the zeta potential of the metallic particles was determined. For the purpose of determining the surface potential of the FeNPs, zeta potential was utilized. In each instance, the stated value was the average of the results of three individual measurements. When the values of the zeta potential ranged from higher than +30 mV to lower than 30 mV, the criteria for the stability of NPs were determined [31].

#### 2.6.6. Particle Size Analyzer

In order to determine the sample’s particle size, PSA testing was performed on it after it had been lyophilized and then dispersed using an ultrasonicator (SZ-100, Horiba, Kyoto, Japan).

#### 2.6.7. X-ray Diffraction Analysis (XRD) Analysis

The synthesized iron nanoparticles from aqueous leaf extract of *V. leucoxylon* were subjected to XRD analysis (Smart Lab SE, Rigaku, Tokyo, Japan)to determine the nature as well as average size of the nanoparticles.

### 2.7. Determination of Antioxidant Activity by Using In Vitro Methods

#### 2.7.1. Ferric-Ion-Reducing Antioxidant Power Assay (FRAP)

According to Oyaizu (1986), with a minor modification, ferric ions’ reducing power was assessed [32]. For 30 min at 50 °C, 2.5 mL of 20 mM phosphate buffer and 2.5 mL of 1% potassium ferricyanide were added to 2.5 mL of *V. leucoxylon* leaf extract and its synthesized FeNPs blended with the combination. Following the incubation period, the mixture was supplemented with 2.5 mL of 10% *w*/*v* trichloroacetic acid and 0.5 mL of 0.1% *w*/*w* ferric chloride before being incubated for an additional 10 min. Finally, a UV-V spectrophotometer was used to detect the absorbance at 700 nm. As a standard, ascorbic acid was utilized. Each sample was tested three times.

#### 2.7.2. Hydrogen Peroxide Scavenging Assay

Based on the ability of *V. leucoxylon* aqueous leaf extract and its synthetic FeNPs to scavenge hydrogen peroxide, the antioxidant activity of these compounds was evaluated. First, 0.6 mL of phosphate buffer (pH—7.4) containing 4 mM H_2_O_2_ was added to 0.5 mL of standard ascorbic acid at a known concentration, as well as tubes containing plant extracts at various concentrations ranging from 100 µL to 500 µL (pH—7.4). Using a phosphate buffer and hydrogen-peroxide-free blank solution, we assessed the solution’s absorbance at 230 nm after 10 min. Phosphate buffer was used to create the control instead of the sample or standard [33]. Each sample was tested three times. The formula approach was used to calculate the inhibition percentage.

Percentage of inhibition % = A_c_ − A_t_/A_c_ × 100

#### 2.7.3. DPPH Free-Radical-Scavenging Assay

*V. leucoxylon* leaf extract and synthesized FeNPs were tested for their ability to scavenge free radicals using DPPH radical as a reagent [34]. Samples were combined with DPPH radical solution (60 M) in ethanol (100 µL) at different concentrations (*w*/*v*). A UV-Vis spectrophotometer was used to measure the absorbance of the mixture at 517 nm after 30 min of incubation in the dark at room temperature. Ascorbic acid was employed as a standard for the experiment. The following equation was used to determine each sample’s DPPH scavenging activity:

% inhibition= A_c_ − A_t_/A_c_ × 100

where A_c_ represents the absorbance of the control reaction, which is performed by mixing 100 L of ethanol with 100 L of the DPPH solution, and A_t_ represents the absorbance of the test sample. Experiments were carried out in triplets. The IC50 value was computed for each sample. A higher level of free radical activity was indicated by the reaction mixture having a lower absorbance.

#### 2.7.4. Phosphomolybdenum (PM) Assay

Total antioxidant activity was determined using the Prieto et al., 1999, standard technique. Each test tube containing 3 mL of distilled water and 1 mL of molybdate reagent solution received aqueous leaf extract of *V. leucoxylon* and its FeNPs in varied concentrations ranging from 100 µL to 500 µL. These tubes were incubated for 90 min at 95 °C. The absorbance of the reaction mixture was measured at 695 nm after these tubes were adjusted to room temperature for 20–30 min after incubation. The reference standard was ascorbic acid [35].

### 2.8. Evaluation of In Vitro Anti-Inflammatory Activity

The anti-inflammatory effect of *V. leucoxylon* aqueous leaf extract and its generated FeNPs was assessed using the protein denaturation method outlined by Elias et al., 1988, with slight modifications [36]. As a standard drug, diclofenac sodium was utilized. A reaction mixture comprising 2 mL of known concentration of manufactured FeNPs (100 g/mL) with standard diclofenac sodium (100 g/mL) and 2.8 mL of phosphate-buffered saline (pH 6.4) was mixed with 2 mL of fresh hen’s egg albumin (1 mM) and incubated at 27 ± 1 °C for 15 min. Denaturation was induced by putting the reaction mixture in a water bath at 70 °C for 10 min. After cooling, the absorbance at 660 nm was measured using double-distilled water as a blank. Each test was carried out three times. The following formula was used to compute the % inhibition of protein denaturation:

% inhibition= A_t_ − A_c_/A_c_ × 100

where, A_t_ = absorbance of test sample; A_c_ = absorbance of control.

### 2.9. Determination of Cytotoxic and Anticancer Activity of Iron Nanoparticles Using MTT Assay

The effect of *V. leucoxylon* and its synthesized FeNPs on the viability of non-cancerous fibroblast cells L292 and its anticancer activity on skin cancer (A375), lung cancer (A549), and oral cancer (KB-3-1) was evaluated using the standard MTT assay, according to Carmichael et al., (1987) [37]. All the cell lines were obtained from the National Centre for Cell Science (NCCS), Pune, India. Percentage inhibition of cell growth (IC50) values were derived using dose-response curves for each cell line, and the following formula was used to compute the percentage growth inhibition. The conversion of MTT to a purple formazan product by healthy cells’ mitochondrial dehydrogenase is the basis of this experiment [38].

Inhibition Percentage = OD of Test sample ÷ OD of control × 100

### 2.10. In Vitro Wound-Healing Study by Using Scratch Assay Test

The spreading and migratory capabilities of L292 cell line cells caused by samples with known concentrations of plant extract and iron nanoparticles were examined in the current investigation [39]. Animal cell culture plates with DMEM media supplemented with 10% FBS and 2% Pen-Strep antibiotic (Darmstadt, Germany) were used to start the cell culture process. A sterile plastic pipette tip was used to scratch the monolayer confluent of cells after they had grown to roughly 50,000 cells per mL. PBS solution was used to remove any unwanted cell debris. As a negative control, untreated cells were used, whereas standard ascorbic acid was used as a positive control for polymer samples of known concentration. For the next 24 h, the cells were kept at 37 °C with 5% CO_2_. For the examination of relative cell migration and wound closure, the scratched cell layers were incubated and imaged at intervals ranging from 0 h to 6 h to 12 h and 24 h. MagVision Software’s measurement (X64, 2016, Magnus, New Delhi, India) calibration at 4× resolution was used to quantify the gap distance. In order to determine the wound closure and migration rate, the formula shown below was used:

Wound closure (%) = A_0h_ − A_Th_/A_Th_ × 100

Rm = W_i_ − W_f_/T

with respect to the following: A_0h_ = wound area measured immediately after scratching; A_Th_ = wound area measured after h hours; Rm = migration rate (µm/h); W_f_ = initial wound width (µm); and T = migration time (hour).

### 2.11. Statistical Analysis

The data are presented as the mean standard deviation and standard error, and each experiment was carried out three times. SPSS software version 20 was used to perform one-way analysis of variance (ANOVA) on the differences in mean scores that existed between the groups.

## 3. Results and Discussion

Nanoparticles (NPs) are particles with a diameter of fewer than 100 nanometers and are essential structural masses in nanotechnology. The most essential characteristic of NPs is that they reveal superior activity [7]. To manufacture various types of NPs, a variety of physical, chemical, biological, and hybrid approaches are now available [7,8,9]. Green synthesis processes have been used to make a variety of metallic nanoparticles, including silver, gold, iron, copper, and zinc. It is a known fact that plant-based phytochemicals act as either reducers or stabilizers in the synthesis of metal nanoparticles and this phyto method is considered to be a safe synthesis approach, producing desirable metal NPs with acceptable structural properties. The high oxidation tendency of phenols, especially in an alkaline condition, can facilitate the initiation of the nucleation process and helps in the reduction of metal salts to metal nanoparticles. In some cases, this oxidized form of phenols attached to the surface of metal nanoparticles and provided stability for the nanoparticles. Natural phenols with functional hydroxyl and carboxyl groups represent protonating and absorbing capabilities. This might be the possible pathway involved in the synthesis of nanoparticles. In light of this, the current research is aimed at FeNPs’ synthesis from aqueous leaf extract of *V. leucoxylon* for biomedical applications.

Preliminary screening entails examining plant crude extracts for the presence of secondary metabolites. Plants are selected among the numerous sources accessible because of their bioreducing and stabilizing capacity. Many biochemical components found in plants, such as alkaloids, flavonoids, tannins, phenols, and saponins, could be used as effective reducing agents in the bioreduction of metal into nanoparticles with a wide range of biological applications. Phenols are non-enzymatic chemicals derived from natural sources that have attracted a lot of attention due to their antioxidant properties. Although phenolic chemicals have been linked to antioxidant activity, certain classes such as flavonoids and tannins have been highlighted in various research studies [40].

In the current study, following biochemical testing, phytochemical screening of aqueous leaf extracts of *V. leucoxylon* revealed the presence of flavonoids, phenols, terpenoids, tannins, and saponins (Table 1). These phytochemicals have been documented for anti-inflammatory, anti-allergic, antibacterial, antiviral, heart failure, antioxidant, and anticancer activities, both traditionally and pharmaceutically [41].

A wide range of scientific disciplines are involved in nanotechnology, including engineering, physics, biology, and medicine [42]. The production of nanoparticles from biological sources, such as fungi and bacteria, is a costly and time-consuming procedure. There are, however, phytosynthesized NPs that do not pose any health dangers [43]. A major advantage of the plant-based approach is that it does not require any expensive procedures such as those required for microbe-based NP synthesis. Anticancer phytochemicals found in medicinal plants have recently been investigated and were pronounced an excellent source for manufacturing natural NPs that pose no health risks and can be produced in huge quantities in a short period of time [13,44].

**Figure 1:**
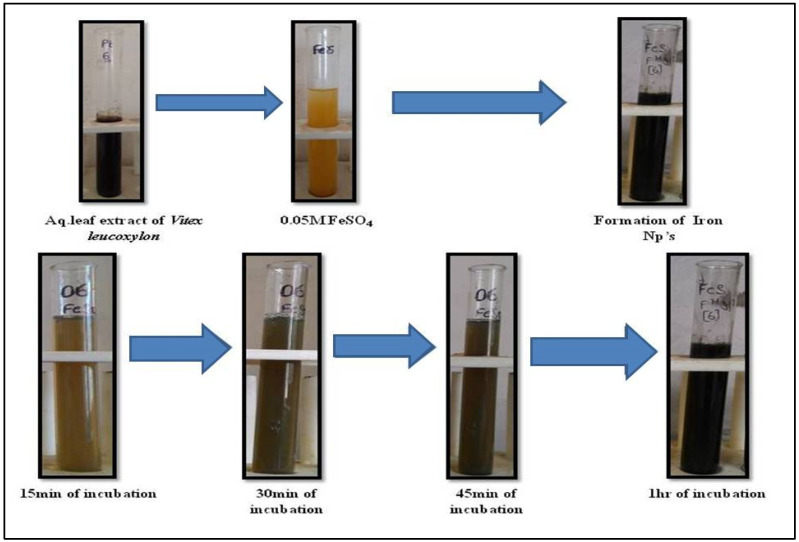
Formation of FeNPs from aqueous leaf extract of *V. leucoxylon* with 0.05 M FeSO_4_.

In the current study, we found that adding an aqueous leaf extract of *V. leucoxylon* to the reaction mixture resulted in a dramatic change in color from yellowish to light black in just 15 min, indicating that the FeSO4 was being bioreduced from iron metal ions Fe+ in solution to FeNPs. After one hour of incubation, the reaction was complete and a dark brown tint had emerged (Figure 1). The UV-Vis spectral measurement of colloidal solution for wavelengths between 200 nm and 700 nm verified the formation of FeNPs. The maximum absorption peak (max) is observed at 395 nm, according to UV-Vis spectral analysis.

**Figure 2:**
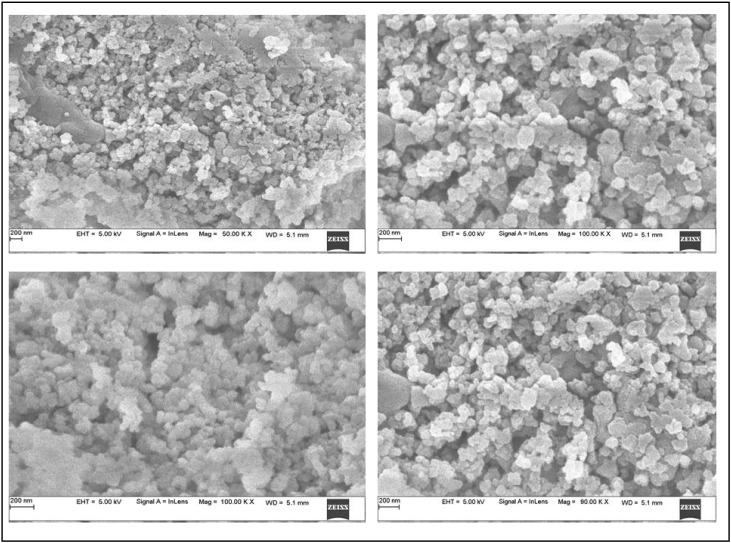
SEM images of spherical-shaped FeNPs.

**Figure 3:**
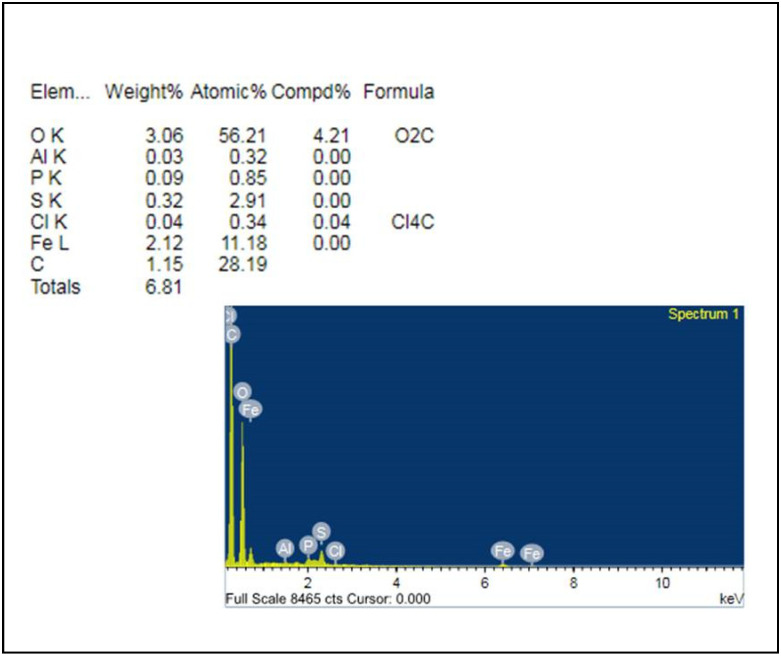
EDX analysis of FeNPs showing its elemental composition.

In the present study, synthesized FeNPs were subjected to structural and elemental confirmation by using SEM and EDX analysis. The FeNPs were found to be spherical in shape, with a diameter ranging from 45 nm to 100 nm, as determined by SEM (Figure 2). The elemental composition of FeNPs was determined by EDX experiments. FeNPs were found to have been formed by the aqueous leaves extract of *V. leucoxylon*, as evidenced by the high signal in the metallic iron region at 6–8 KeV. Its elemental composition studies show that it contains a variety of other elements such as carbon, aluminum, and silicon as well as iron as the primary element with 11.18% (Figure 3).

**Figure 4:**
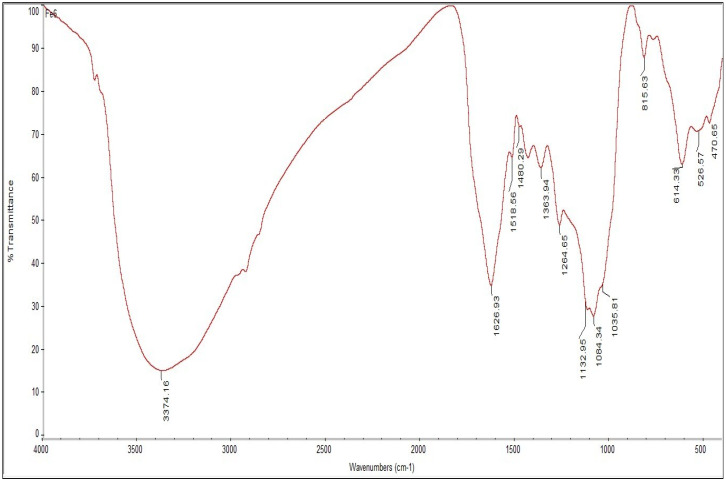
FTIR spectra showing the peaks of FeNPs synthesized from *V. leucoxylon*.

**Figure 5:**
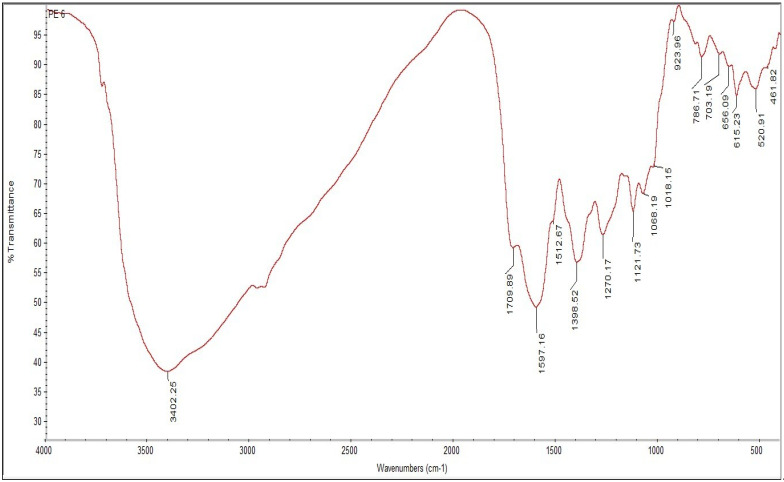
FTIR spectra showing the peaks of aqueous leaf extracts of *V. leucoxylon*.

The functional groups responsible for phytoreduction of Fe^+^ into FeNPs were identified using FTIR spectroscopy. Figure 4 and Figure 5 show the FTIR spectra of FeNPs and plant extract, respectively. The FTIR spectra revealed shifts in some peaks of *V. leucoxylon* aqueous leaf extract, namely 923.96 cm^−1^ and 1709.89 cm^−1^, with functional groups carboxylic acids, unsaturated aldehydes, and ketones, which were lacking in the FTIR spectra of FeNPs, and were responsible for FeNPs formation. However, some functional groups were present in both FTIR spectra of plant extract and FeNPs, such as alkyl halides, alkynes, aromatics and aliphatic amines, esters, ethers, alcohol, carboxylic acids, and nitro compounds with peaks at 526.57 cm^−1^, 614.33 cm^−1^, 815.63 cm^−1^, 1035.81 cm^−1^, 1084.34 cm^−1^, 1132.95 cm^−1^, and 1264 cm^−1^.

**Figure 6:**
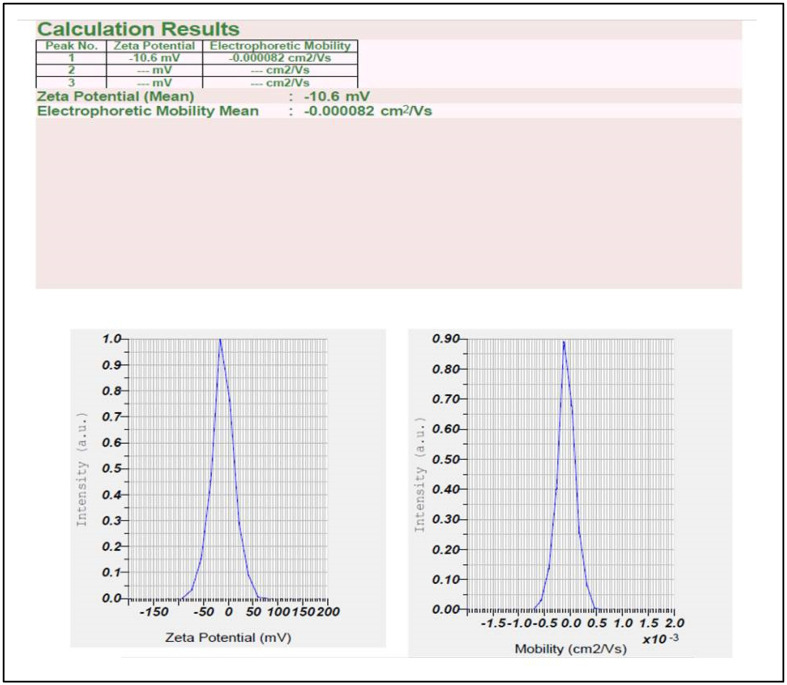
Zeta potential distribution of *V. leucoxylon* FeNPs.

**Figure 7:**
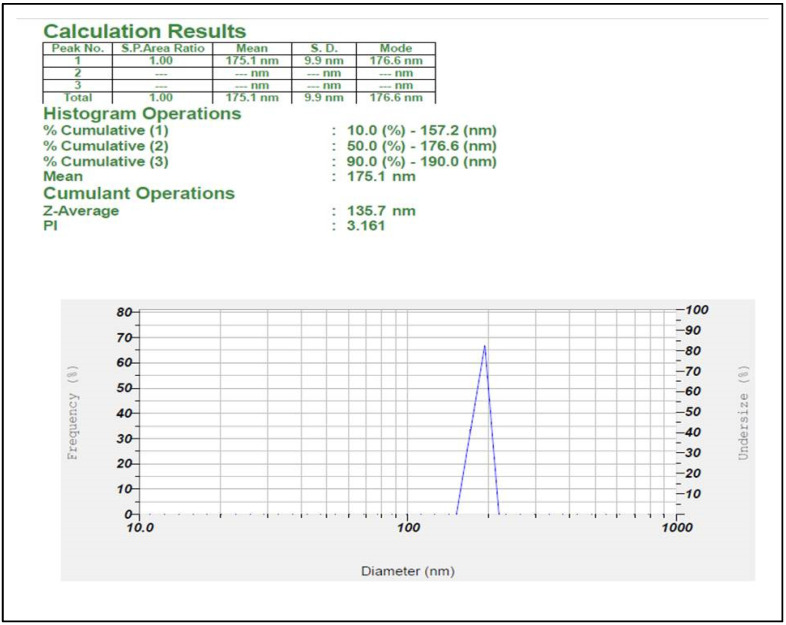
Analysis of particle size of *V. leucoxylon* FeNPs.

Further, the iron nanoparticles were subjected to the study of stability and particle size analyzer by using zeta potential and particle size analyzer. Results showed that synthesized iron nanoparticles were quite stable with negative value i.e., −10.6 mV (Figure 6), whereas the formed iron nanoparticles have shown an average size of 135.2 nm (Figure 7).

**Figure 8:**
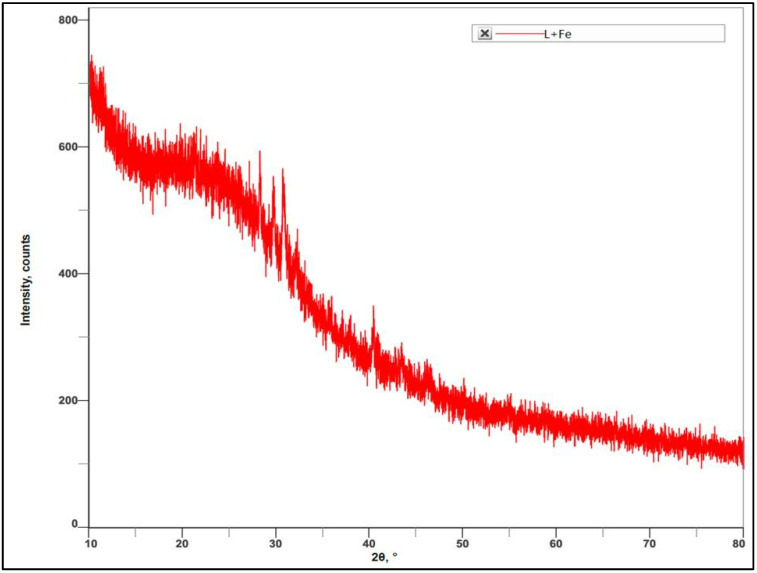
XRD analysis of *V. leucoxylon* FeNPs.

Furthermore, the XRD analysis results showed the sharp peaks at different angles, i.e., 21.87, 29.85, 30.76, 40.40, 43.42, and 46.18. These degrees of angles correspond to the crystalline nature of the material. Hence, it was confirmed that the synthesized iron nanoparticles were crystalline in nature. XRD analysis also confirmed that the synthesized nanoparticles were in nano size with average diameter of 136.43 nm (Figure 8).

Because of their sophisticated properties, nanoparticles have become widely used in a variety of fields in recent years. They are favored because of their high surface area, low toxicity, and ease of separation. Iron oxide nanoparticles less than 20 nm in size, such as maghemite or magnetite, have distinct characteristics [45]. They are employed in biomedical applications to separate and purify cell populations for diagnostic magnetic resonance imaging (MRI), medication administration to a cell or tissue, and cell biology research [46].

Free radicals are highly reactive unstable atoms or molecules with unpaired outermost electrons that are produced by reactive oxygen species (ROS). These are to blame for a variety of human ailments such as atherosclerosis and cancer, as well as brain damage and persistent difficulties in the physiological system. Oxidative stress is a major contributor to degenerative senescence. ROS has been linked to the pathophysiology of a variety of biological processes, as well as a number of diseases such as cardiovascular, cancer, neurological, and respiratory diseases [47]. 

In the current study, both aqueous leaf extract of *V. leucoxylon* and its FeNPs were subjected to in vitro antioxidant research using various assays such as FRAP, H_2_O_2_, DPPH, and PM assays. The FRAP assay employs ferricyanide and ferric ions as chromogenic oxidants. It provides an immediate result for a wide range of individual antioxidants in a dose-dependent manner, with color intensity directly proportionate to antioxidant reduction potency. The hue of the reaction mixture at 700 nm and higher absorbance implies increased antioxidant activity. 

**Figure 9:**
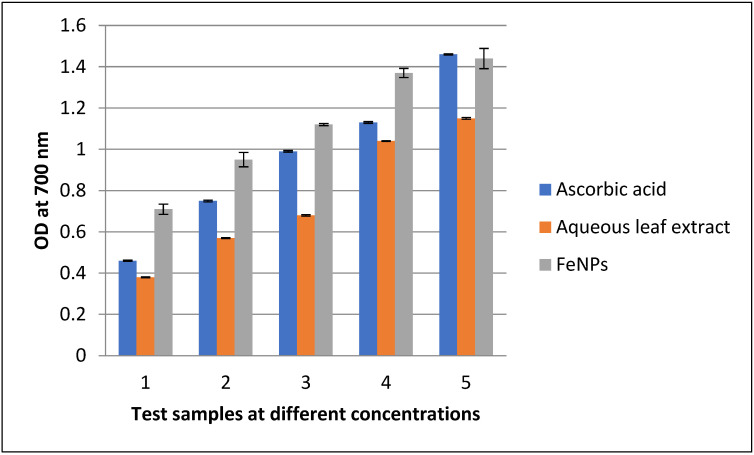
FRAP assay of aqueous leaf extract of *V. leucoxylon* and its synthesized FeNPs.

In the current investigation, known quantities of aqueous leaf extracts of *V. leucoxylon* and it FeNPs were tested using the FRAP assay with ascorbic acid as the standard. Increased concentrations of both standard and FeNPs result in increased antioxidant activity and absorption. The antioxidant activity of FeNPs was higher when compared to aqueous leaf extracts of *V. leucoxylon*, whereas the standard had the highest activity. (Table 2 and Figure 9).

One of the most used methods for determining the antioxidant capacity of extracts is the H_2_O_2_ test. Although hydrogen peroxide is relatively passive, it can have harmful effects on cells by producing hydroxyl radicals and making them poisonous. H_2_O_2_ can be eliminated by antioxidants such as phenols, polyphenols, and flavonoids, which protect mammalian cells from hydrogen peroxide damage [48]. The scavenging activity of extracts was determined in this experiment and compared to ascorbic acid, which served as the standard. The assay revealed that FeNPs showed higher activity than plant extract, with percentage of inhibition, i.e., 74.46 ± 0.130, whereas plant extract showed 69.76 ± 0.427 and standard ascorbic acid showed the highest activity with 80.86 ± 0.155 of inhibition (Table 3).

**Figure 10:**
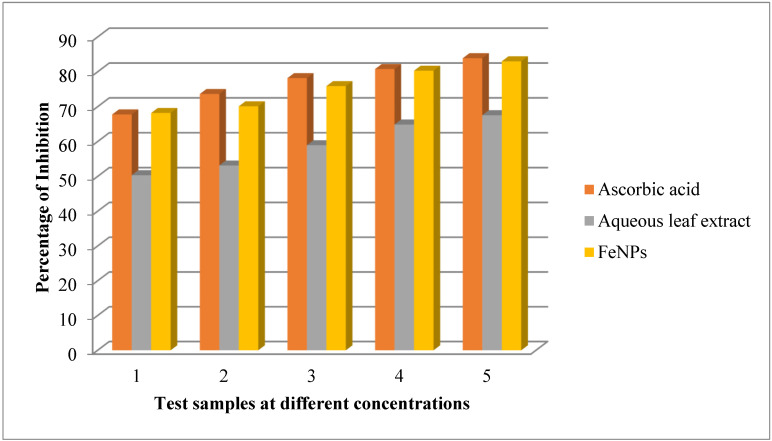
DPPH assay of aqueous leaf extract of *V. leucoxylon* and its synthesized iron nanoparticles.

The DPPH assay is one of the most extensively used methods for determining antioxidant activity in a variety of plant-based medicines and phytochemical constituents [49]. It works by using free radical scavengers to reduce a methanolic solution of colored free radical DPPH. Free radical scavenger concentration is proportional to DPPH scavenging absorbance at 517 nm. The test extracts’ reducing potential is assessed by a decrease in absorbance. The antioxidant activity of FeNPs was compared to that of a conventional antioxidant, ascorbic acid. The antioxidant activity of produced FeNPs was lower than that of standard, whereas aqueous leaf extracts of *V. leucoxylon* showed superior inhibition, which was comparable to that of standard ascorbic acid (Table 4 and Figure 10). 

**Figure 11:**
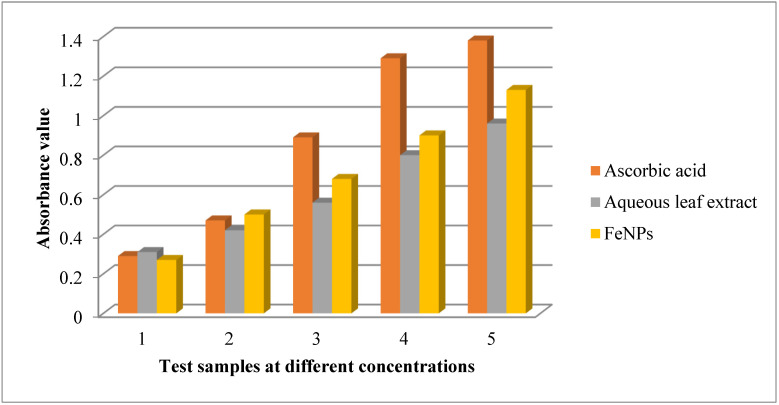
PM assay of aqueous leaf extract of *V. leucoxylon* and its synthesized iron nanoparticles.

Redox reactions involving molybdenum and molybdenum ligand antioxidants can be quantified using the PM test. It entails a longer duration of incubation at a higher temperature, which alters the auto-oxidation reaction in the mixture. It directly measures the extracts’ lowering potency. At acidic pH, the reaction mixture creates a green phosphomolybdeum colored complex, which is detected at 695 nm. The reducing activity of extracts and conventional drugs increased as concentrations increased. In the current investigation, known amounts of aqueous leaf extracts of *V. leucoxylon* and its generated FeNPs, as well as ascorbic acid as a standard, were exposed to PM screening. Iron nanoparticles demonstrated greater action than aqueous leaf extracts of *V. leucoxylon*, while conventional ascorbic acid demonstrated the highest activity. (Table 5 and Figure 11). 

Inflammation is the immunological reaction to a specific event, whereas infection is the term used to describe pathogenic invasion. When the body tries to protect itself, inflammation arises. Injury, illness, and stress cause inflammation, which is a localized phenomenon. Anti-inflammatory medicines, both steroidal and non-steroidal, are commonly used to treat inflammation; however, the treatments’ side effects can be harmful [50]. Anti-inflammatory medications have been developed using nano-based herbal formulations. Numerous studies have emphasized the anti-inflammatory benefits of metallic NPs produced from plant extracts.

The present study used an in vitro protein denaturation experiment with known doses of aqueous leaf extracts of *V. leucoxylon* and its generated FeNPs to investigate anti-inflammatory effects. The samples’ anti-inflammatory effect in vitro was comparable to that of diclofenac sodium, a standard medication. Within the group, there was a significant variation in protein denaturation. The results demonstrated that FeNPs had strong anti-inflammatory efficacy, with a percentage of inhibition of 78.84% versus 67.65% for aqueous leaf extracts of *V. leucoxylon*, and 89.64% for standard medication diclofenac sodium (Table 6).

**Figure 12:**
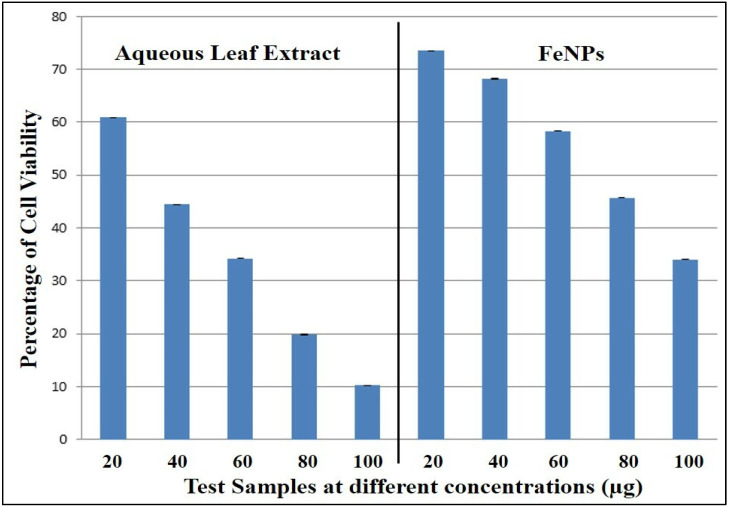
Percentage of cell viability of aqueous leaf extract of *V. leucoxylon* and its synthesized iron nanoparticles against L929 cell line.

**Figure 13:**
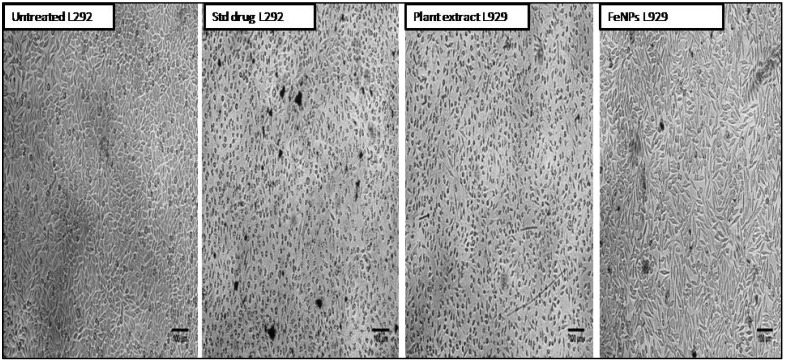
Morphological study of effect of aqueous leaf extract of *V. leucoxylon* and its synthesized iron nanoparticles against L929 cell line. The scale used is 100 µm.

Further the synthesized iron nanoparticles were subjected to the study of toxicity based on MTT cell viability assay by using non-cancerous fibroblast cell line L929. The present cytotoxicity cell viability assay revealed that in both tested samples, dose-dependent activity was observed. In comparison, the aqueous leaf extract of *V. leucoxylon* has shown a greater decrease in cell viability compared to the FeNPs-treated cells. In the case of plant extract, the percentage of cell viability at 100 µg concentration was observed to be 10.23 ± 0.012, whereas, for FeNPs it was observed to be 34.06 ± 0.001 (Table 7 and Figure 12 and Figure 13). 

The survival and proliferation of malignant cancer cells are defined by anomalies in systems that regulate the cell cycle in cancer. When cancer develops, a wide range of signaling pathways are affected. Cancer formation and radiation and chemotherapy resistance are both facilitated by inhibiting natural apoptosis [51]. Cells divide rapidly and continuously in cancer, making it a non-communicable disease [52]. A lack of proper diagnosis and therapy has been blamed for the high expense of cancer care and cancer theranostics [53]. In terms of cancer fatalities, lung, breast, and colorectal cancers have the highest incidence rates of all cancers. Surgery, chemotherapy, radiation, immunotherapy, and hormonal therapy are among the options available for treatment. Despite the fact that these methods are often used today, they have been linked to a number of adverse consequences, ranging from the mild to the severe [52].

The most prevalent type of head and neck cancer is oral cancer. People over 60 are typically affected. It is one of the top 10 malignancies in terms of occurrence, and despite advances in research and treatment, survival rates have not increased noticeably in recent years, posing a persistent problem for biomedical science. Use of tobacco products, binge drinking, and human papillomavirus infection are all risk factors. A sore that does not heal, a lump, or a white or red area on the inside of the mouth are typical symptoms. Radiation therapy, chemotherapy, and surgery are used as treatments [54]. 

Lung cancer is one of the deadliest and most aggressive cancers, capable of spreading throughout the body and causing a great deal of mortality. Males and females both are affected by this cancer, which is the third most lethal of the three major malignancies [55]. Loss of weight, weariness, coughing up blood, and dysphagia are some of the most prevalent symptoms of lung cancer [56,57]. Treatment options for people with lung cancer include chemotherapy, radiation therapy, laser therapy, immunotherapy, and surgery [58]. As a result of side effects such as nerve damage, toxicity, hair loss, and exhaustion as well as diarrhea and mouth sores, the researchers have been looking for novel compounds; it has been a major concern for them [59].

**Figure 14:**
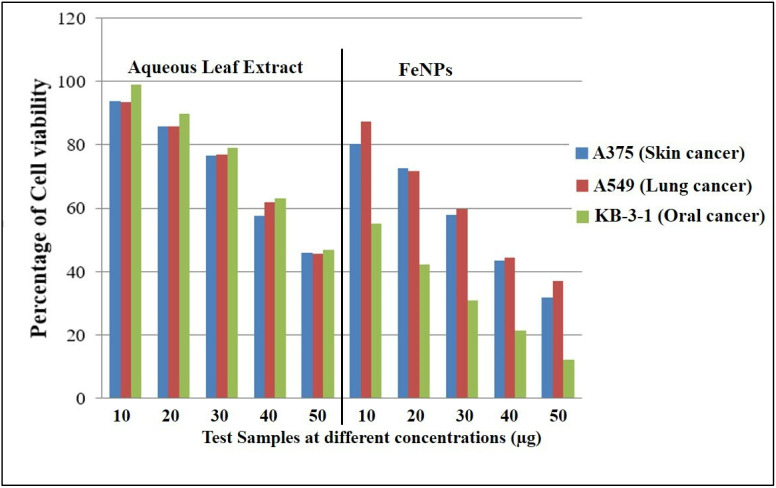
Percentage of cell viability for aqueous leaf extract of *V. leucoxylon* and its synthesized iron nanoparticles against different cancer cell lines.

**Figure 15:**
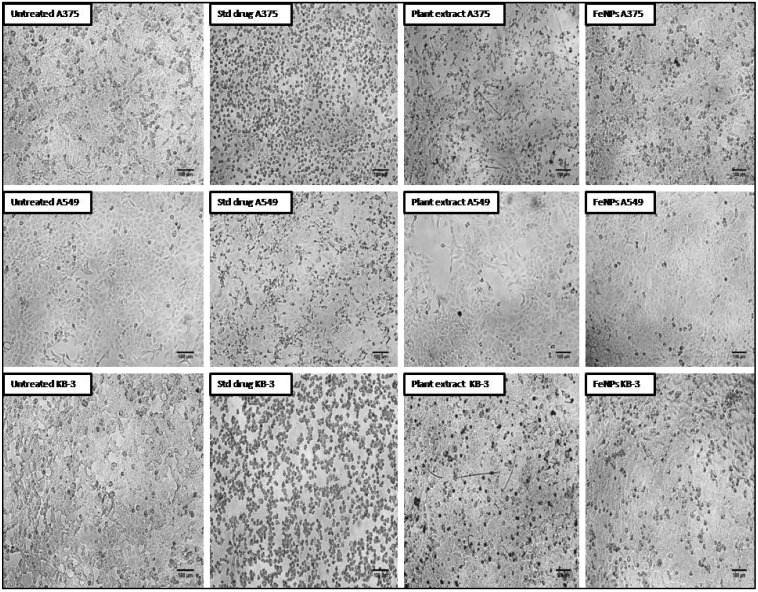
Morphological study of effect of aqueous leaf extract of *V. leucoxylon* and its synthesized iron nanoparticles against different cancer cell lines. The scale used is 100 µm.

In the current study, the aqueous leaf extract of *V. leucoxylon* and its FeNPs were subjected to evaluating cytotoxic activity by testing against skin cancer (A375), lung cancer (A549), and oral cancer (KB-3-1). The results revealed that FeNPs have shown significant activity, with high percentage of cell death and low percentage of cell viability. On comparison within the tested group of cancer cell lines, FeNPs have shown higher activity in oral cancer, followed by skin cancer and lung cancer. Compared to FeNPs, the plant extract MTT cell viability results showed moderate activity (Table 8, Figure 14 and Figure 15). 

A wound is an injury to the skin that causes the dermal layers to be sliced, pierced, or torn as a result of stressors or trauma [60]. Acute and chronic wounds are generally divided into two categories based on healing time and other factors. Chronic wounds (venous, diabetic foot, or pressure ulcers) are becoming increasingly common, reaching epidemic proportions, justifying the rising interest in discovering more effective treatments [61]. The complexity of the normal wound-healing process, cell type specificity, and plethora of regulatory chemicals, as well as the pathophysiology of chronic wounds, are all targets for nanotechnology-based diagnostics and therapeutic techniques. The use of nanotherapeutics in cutaneous wound healing has significant theoretical advantages. Burn burns are the most dangerous type of trauma, and infection control is essential to limit morbidity and mortality [62]. Burn patients are sensitive to infection problems because severe thermal injury damages the skin’s surface barrier and causes an immunological condition [63]. Negative pressure wound therapy, hyperbaric oxygen therapy, bioengineered cell constructs, dressing materials, and vascular surgery are some of the current treatments. Nonsteroidal pharmaceuticals (ibuprofen, naproxen, rofecoxib, and celecoxib) and chemotherapeutic drugs (cabozantinib, bevacizumab, lenvatinib, refametinib, brivanib, and everolimus) are routinely used in addition to glucocorticoids. However, these medications all have a variety of side effects that limit their use.

**Figure 16:**
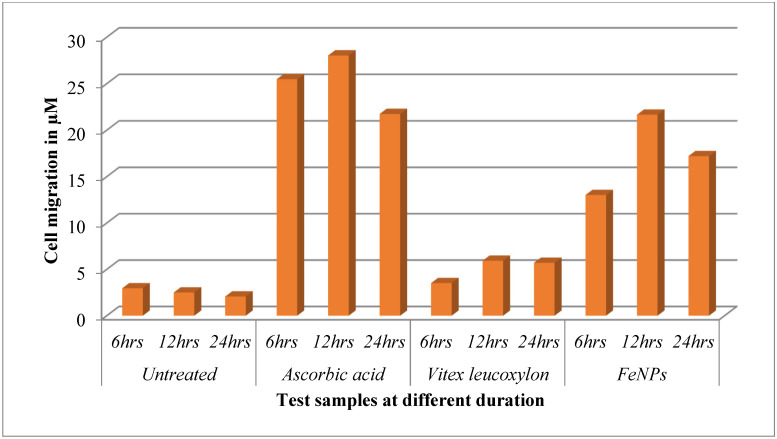
Cell migration of *V. leucoxylon* extract and its synthesized FeNPs.

**Figure 17:**
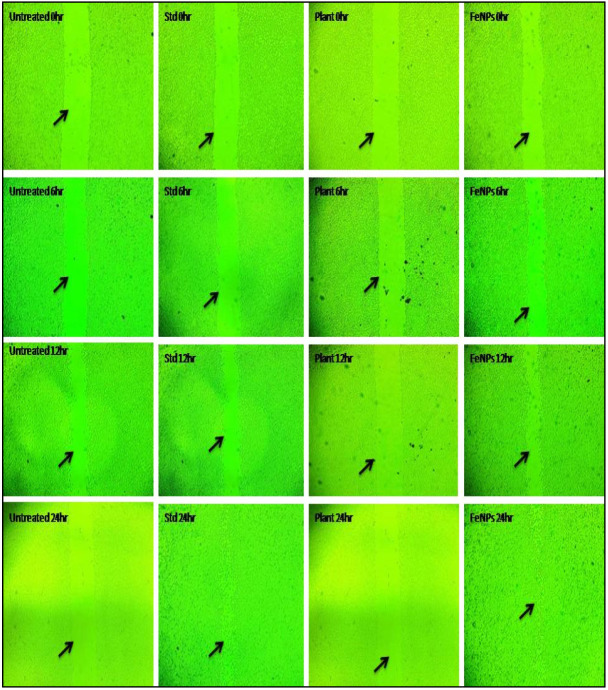
Morphological wound closure studies of *V. leucoxylon* and its synthesized iron nanoparticle. Arrows indicate cell migration for wound healing.

In the current study, the FeNPs and aqueous leaf extract of *V. leucoxylon* were subjected to the study of wound-healing activity by following cell migration, as well as wound closure study using scratch assay with normal fibroblast L929 cell line. Cell migration results showed that there is increase in the cell migration in standard and FeNPs-treated cells when compared to aqueous leaf extract of *V. leucoxylon*. In comparison, standard drug ascorbic acid showed higher cell migration activity, followed by FeNPs, plant extract, and untreated. (Table 9 and Figure 16). In the case of the wound closure study, the 24 h of incubation was considered for the analysis and calculation. The standard drug ascorbic acid showed the highest percentage of wound closure followed by FeNPs, plant extract, and untreated (Table 10 and Figure 17)

## 4. Conclusions

The aqueous leaf extract of *V. leucoxylon* was used to successfully synthesize iron nanoparticles. The leaf extract’s biomolecules were responsible for FeNPs production and stability. UV-Vis spectroscopy, SEM, XRD, and EDX were used to characterize size, morphology, crystalline structure, and stability. FTIR evaluated FeNPs’ functional groups. Gallic acid may reduce FeSO4 to generate FeNPs in leaf extract. Adding aqueous leaf extract of *V. leucoxylon* to 0.05 M FeSO_4_ caused a color shift from yellowish to black within 1 h. UV-Vis spectral analysis of colloidal solution for wavelength scanning between 200–700 nm showed maximal absorption peak (max) at 395 nm. SEM scans indicated 45–100 nm spherical FeNPs. EDX measurements show a high signal of metallic iron at 6–8 Kev, confirming the synthesis of FeNPs with 11.18% purity. FTIR spectroscopy demonstrated shifts in some peaks of aqueous leaves extract of *V. leucoxylon*, i.e., 923.96 cm^−1^, 1709.89 cm^−1,^ with carboxylic acids, unsaturated aldehydes, and ketones. Iron nanoparticles are potent in antioxidant, anti-inflammatory, cytotoxic, and wound-healing activities. 

## Figures and Tables

**Table 1:** Phytochemical analysis of aqueous leaf extract of *V. leucoxylon*.

Tests	Water Extract
Alkaloids	−ve
Flavonoids	+ve
Glycosides	−ve
Phenols	+ve
Saponins	+ve
Tannins	−ve
Terpenoids	+ve
Steroids	−ve

+ ve: presence; − ve: absence.

**Table 2:** FRAP assay of aqueous leaf extract of *V. leucoxylon* and its synthesized iron nanoparticles.

Sl.no	Concentration in µg	Ascorbic acid	Aqueous leaf extract	FeNPs
1	100	0.46 ± 0.003	0.38 ± 0.003	0.71 ± 0.025
2	200	0.75 ± 0.004	0.57 ± 0.002	0.95 ± 0.035
3	300	0.99 ± 0.004	0.68 ± 0.004	1.12 ± 0.005
4	400	1.13 ± 0.004	1.04 ± 0.002	1.37 ± 0.022
5	500	1.46 ± 0.003	1.15 ± 0.004	1.44 ± 0.049

Results are expressed as mean ± SD (*n* = 3).

**Table 3:** H_2_O_2_ assay of aqueous leaf extract of *V. leucoxylon* and its synthesized iron nanoparticles.

Sl.no	Concentration in µg	Samples	Percentage of Inhibition
1	100	Ascorbic Acid	80.86 ± 0.155
2	100	Aqueous Leaf Extract	69.76 ± 0.427
3	100	Iron Nanoparticles	74.46 ± 0.130

Results are expressed as mean ± SD (*n* = 3).

**Table 4:** DPPH assay of aqueous leaf extract of *V. leucoxylon* and its synthesized iron nanoparticles.

Sl.no	Concentration in µg	Ascorbic Acid	Aqueous Leaf Extract	FeNPs
1	10	67.78 ± 0.178	50.34 ± 0.003	68.21 ± 0.025
2	20	73.65 ± 0.235	53.13 ± 0.002	70.11 ± 0.035
3	30	78.20 ± 0.358	58.95 ± 0.004	75.90 ± 0.005
4	40	80.80 ± 0.356	64.88 ± 0.002	80.33 ± 0.022
5	50	83.91 ± 0.350	67.55 ± 0.004	83.02 ± 0.049

Results are expressed as mean ± SD (*n* = 3).

**Table 5:** PM assay of aqueous leaf extract of *V. leucoxylon* and its synthesized iron nanoparticles.

Sl.no	Concentration in µg	Ascorbic Acid	Aqueous Leaf Extract	FeNPs
1	100	0.29 ± 0.002	0.31 ± 0.016	0.27 ± 0.025
2	200	0.47 ± 0.001	0.42 ± 0.031	0.50 ± 0.035
3	300	0.89 ± 0.002	0.56 ± 0.044	0.68 ± 0.005
4	400	1.29 ± 0.002	0.80 ± 0.097	0.90 ± 0.022
5	500	1.38 ± 0.002	0.96 ± 0.033	1.13 ± 0.049

Results are expressed as mean ± SD (*n* = 3).

**Table 6:** In vitro anti-inflammatory effect of aqueous leaf extract of *V. leucoxylon* and its synthesized iron nanoparticles.

Sl.no	Concentration in µg	Samples	Percentage of Inhibition
1	100	Ascorbic Acid	89.64 ± 0.042
2	100	Aqueous Leaf Extract	67.65 ± 0.053
3	100	Iron Nanoparticles	78.84 ± 0.030

Results are expressed as mean ± SD (*n* = 3).

**Table 7:** Percentage of cell viability of aqueous leaf extract of *V. leucoxylon* and its synthesized iron nanoparticles against L929 cell line.

Samples	Concentration in µg	Percentage of Cell Viability	IC_50_ in µg
Aqueous leaf extract of *V. leucoxylon*	20	60.89 ± 0.004	34.51
	40	44.44 ± 0.003	
	60	34.24 ± 0.001	
	80	19.82 ± 0.006	
	100	10.23 ± 0.012	
FeNPs	20	73.49 ± 0.001	71.71
	40	68.21 ± 0.005	
	60	58.28 ± 0.001	
	80	45.68 ± 0.005	
	100	34.06 ± 0.001	

Results are expressed as mean ± SE (*n* = 3).

**Table 8:** Percentage of cell viability for aqueous leaf extract of *V. leucoxylon* and its synthesized iron nanoparticles against different cancer cell lines.

Samples	Concentration in µg	Percentage of Cell Viability for A375	Percentage of Cell Viability for A549	Percentage of Cell Viability for KB-3-1
Aqueous leaf extract of *V. leucoxylon*	10	93.80 ± 0.004	93.53 ± 0.001	99.12 ± 0.002
	20	85.78 ± 0.003	85.81 ± 0.003	89.85 ± 0.005
	30	76.69 ± 0.005	76.92 ± 0.001	78.99 ± 0.001
	40	57.49 ± 0.001	61.97 ± 0.002	63.04 ± 0.005
	50	46.00 ± 0.004	45.59 ± 0.001	46.96 ± 0.001
FeNPs	10	80.24 ± 0.003	87.41 ± 0.025	55.25 ± 0.001
	20	72.66 ± 0.001	71.63 ± 0.001	42.23 ± 0.002
	30	57.89 ± 0.002	59.66 ± 0.001	30.99 ± 0.021
	40	43.38 ± 0.002	44.48 ± 0.003	21.47 ± 0.003
	50	31.79 ± 0.002	37.16 ± 0.006	12.14 ± 0.002

Results are expressed as mean ± SE (*n* = 3).

**Table 9:** Cell migration of different test samples at different duration.

Sl.No	Test sample	Duration	Cell Migration in µm
1	Untreated	6 h	2.96
		12 h	2.50
		24 h	2.07
2	Ascorbic acid	6 h	25.47
		12 h	28.03
		24 h	21.74
3	*Vitex leucoxylon*	6 h	3.52
		12 h	5.93
		24 h	5.71
4	FeNPs	6 h	13.02
		12 h	21.66
		24 h	17.21

**Table 10:** Percentage of wound closure of different test samples.

Sl.No	Test Sample	Percentage (%) of Wound Closure at 24 h
1	Untreated	9.13
2	Standard drug Ascorbic acid	96.26
3	*Vitex leucoxylon*	25.25
4	FeNPs	93.71

## Data Availability

The data related to this research are included in the Results section.
